# The Influence of Greenspace Exposure on Affect in People With and Those Without Schizophrenia: Exploratory Study

**DOI:** 10.2196/44323

**Published:** 2023-08-03

**Authors:** Tairmae Kangarloo, Jasmine Mote, Samuel Abplanalp, Alisa Gold, Peter James, David Gard, Daniel Fulford

**Affiliations:** 1 Sargent College of Health and Rehabilitation Sciences Boston University Boston, MA United States; 2 Desert Pacific Mental Illness Research, Education and Clinical Center Veterans Affairs Greater Los Angeles Healthcare System Los Angeles, CA United States; 3 Department of Psychiatry and Biobehavioral Sciences University of California, Los Angeles Los Angeles, CA United States; 4 Harvard Medical School and Harvard Pilgram Health Care Institute Boston, MA United States; 5 Harvard TH Chan School of Public Health Boston, MA United States; 6 Department of Psychology San Francisco State University San Francisco, CA United States; 7 Department of Psychological and Brain Sciences Boston University Boston, MA United States

**Keywords:** greenspace, mental health, mobile technology, affect, smartphone, sensing, schizophrenia, natural vegetation, mental health, exposure, assessment, mechanism

## Abstract

**Background:**

Exposure to natural vegetation (ie, “greenspace”) is related to beneficial outcomes, including higher positive and lower negative affect, in individuals with and those without mental health concerns. Researchers have yet to examine dynamic associations between greenspace exposure and affect within individuals over time. Smartphone-based ecological momentary assessment (EMA) and passive sensors (eg, GPS, microphone) allow for frequent sampling of data that may reveal potential moment-to-moment mechanisms through which greenspace exposure impacts mental health.

**Objective:**

In this study, we examined associations between greenspace exposure and affect (both self-reported and inferred through speech) in people with and those without schizophrenia spectrum disorder (SSD) at the daily level using smartphones.

**Methods:**

Twenty people with SSD and 14 healthy controls reported on their current affect 3 times per day over 7 days using smartphone-based EMA. Affect expressed through speech was labeled from ambient audio data collected via the phone’s microphone using Linguistic Inquiry and Word Count (LIWC). Greenspace exposure, defined as the normalized difference vegetation index (NDVI), was quantified based on continuous geo-location data collected from the phone’s GPS.

**Results:**

Overall, people with SSD used significantly more positive affect words (*P*=.04) and fewer anger words (*P*=.04) than controls. Groups did not significantly differ in mean EMA-reported positive or negative affect, LIWC total word count, or NDVI exposure. Greater greenspace exposure showed small to moderate associations with lower EMA-reported negative affect across groups. In controls, greenspace exposure on a given day was associated with significantly lower EMA-reported anxiety on that day (*b*=–0.40, *P*=.03, 95% CI –0.76 to –0.04) but significantly higher use of negative affect words (*b*=0.66, *P*<.001, 95% CI 0.29-1.04). There were no significant associations between greenspace exposure and affect at the daily level among participants with SSD.

**Conclusions:**

Our findings speak to the utility of passive and active smartphone assessments for identifying potential mechanisms through which greenspace exposure influences mental health. We identified preliminary evidence that greenspace exposure could be associated with improved mental health by reducing experiences of negative affect. Future directions will focus on furthering our understanding of the relationship between greenspace exposure and affect on individuals with and those without SSD.

## Introduction

The potential health benefits of spending time in nature have been of interest to researchers and health professionals for centuries. Research has shown that viewing pictures and videos of nature was associated with higher self-reported positive affect and lower negative affect and physiological markers of stress (eg, muscle tension) than viewing urban areas [1]. Continued work has explored the benefits of nature in daily life across various populations from college students [2] to army medics [3], and across different stimuli, including watching videos of nature [4] or experiencing nature walks using virtual reality [5]. These studies explored not only the restorative properties of greenspace but showed that decreased exposure is associated with lower quality of life [6-8].

Greenspace refers to grass, trees, and other vegetation in a given area. Exposure to greenspace can be defined in a number of ways, but generally refers to the amount of contact or access that individuals have to natural, outdoor environments such as parks, forests, or gardens [9,10]. Several methods can be used to measure greenspace. Geo-location data can be used to map and quantify the amount of greenspace within a defined area, while field surveys can provide detailed information about the characteristics of greenspace. A proxy for better understanding greenspace in a given geographical area is the normalized difference vegetation index (NDVI), a scale used by researchers to quantify the density of vegetation. Previous studies have used these methods to investigate the relationship between greenspace exposure and health [11,12].

Exposure to greenspace has been linked to improved mental health [13]. In a retrospective study in the Netherlands using electronic medical records, people who lived in areas with more access to greenspace reported lower levels of anxiety and depression compared to those in urban areas, especially in people with lower incomes [14]. In a longitudinal study in the United Kingdom, people who moved to an area with more greenspace showed improvements in self-reported mental health from before to 3 years after the move [15]. Greenspace may also confer protective benefits on mental health. In a study of children living in rural areas, the impact of stress on well-being was lower in those with greater access to nature [16]. In sum, greenspace appears to be associated with better mental health in the general population.

Studies among those diagnosed with mental illness, including people with schizophrenia spectrum disorder (SSD), may provide further insight regarding the mechanisms through which greenspace exposure impacts mental health and well-being [17]. There is a large body of work demonstrating an association between psychosis and living in urban environments, which typically afford fewer opportunities for greenspace exposure [18]. Indeed, people with SSD are less likely to live in areas with access to greenspace [19], a potential consequence of lower educational attainment [20] and lower rates of employment [21] relative to the general population. People with SSD may also be less likely to spend time in outdoor areas, such as parks or forests, given limited resources, as well as other challenges such as limited motivation [22] and smaller social networks [23]. Urban areas also have other attributes that are detrimental to health, including overcrowding and air pollution.

Only 2 studies have directly examined the relationship between greenspace exposure and the incidence of serious mental illness, with mixed results. In one study, more greenspace exposure during the first decade of life was associated with a lower incidence of psychosis in adulthood [24]. In contrast, in a retrospective study of health registry data in the United Kingdom, rates of serious mental illnesses were higher in areas with greater tree density [25]. As such, evidence of an association between greenspace exposure and the incidence of serious mental illness is mixed.

Ambulatory assessments supported by mobile technologies may help clarify the relationship between greenspace exposure and mental health in those with and without mental illness. The ubiquity of smartphones has allowed for the feasible use of ecological momentary assessment (EMA), or the experience sampling method, to actively collect data on individual experiences and behaviors repeatedly over days, weeks, or months in the context of daily life. Furthermore, data can be collected passively and semicontinuously using smartphone sensors, supplementing EMA surveys [26]. Among these sensors, geo-location derived from GPS may be especially useful for quantifying greenspace exposure, allowing for spatial and temporal data specific to an individual’s location over time [27]. Other passive data could include audio collected from the phone’s microphone to characterize affect or other features of participants’ speech as a means of supplementing self-reported experiences with affect expression [28].

The combination of passive and active smartphone data can provide a comprehensive understanding of potential correlates of greenspace exposure, ultimately informing our understanding of the impact of greenspace on mental health and well-being. In one recent ambulatory study among people with SSD [29], greenspace exposure (aggregated across the sampling period) was associated with lower overall EMA-reported anxiety, depression, and psychosis symptoms. While promising, the findings of this study could not speak to the degree to which changes in greenspace exposure might covary with affect experience within individuals over time (eg, at the daily level), using both active and passive smartphone data.

In this study, we examined daily associations between greenspace exposure and affect in people with SSD and a comparison group with no known mental illness using smartphones. We measured affect using both active and passive data streams (ie, speech through the smartphone’s microphone), and measured greenspace exposure using NDVI values spatially joined with GPS-based geo-location. Given the exploratory nature of the work, and limited sample size, we primarily aimed to assess the utility of using smartphone-based active and passive measures to understand associations between greenspace and its affect in daily life. Specifically, we hypothesized that controls would have significantly greater mean NDVI throughout the study than people with SSD. Additionally, we predicted that within groups, greater exposure to greenspace on a given day would be associated with (1) higher ratings of positive affect (happiness) and lower ratings of negative affect (anxiety and sadness), and (2) greater use of positive affect words and lower use of negative affect words on that same day.

## Methods

This study involves a secondary analysis of data collected as part of another study. Methods are described in detail in the primary outcomes paper.

### Recruitment

Twenty people with SSD and 15 controls (no psychiatric diagnosis) were recruited from the San Francisco Bay Area in 2016. Community-based participants with SSD were recruited via outpatient clinician referrals and brochures or flyers posted in local clinics. Control participants were recruited via community flyers and public advertisements on websites typically used to recruit for psychological research studies. SSD participants were eligible for the study if they met “Diagnostic and Statistical Manual of Mental Disorders, Fourth Edition” criteria for a diagnosis of SSD using the Structured Clinical Interview for Diagnostic and Statistical Manual of Mental Disorders, Fourth Edition [30]. Controls were eligible if they did not have a past or current history of Axis I disorders as characterized by the Structured Clinical Interview for Diagnostic and Statistical Manual of Mental Disorders, Fourth Edition. All participants had to be between the ages of 18 and 70 years, be fluent in English, and not have a past history of head trauma, stroke, neurological disease, or loss of consciousness. Participants were excluded if they had a current mood episode (depression or mania) or substance dependence within the past 6 months.

Potential participants completed a brief phone screening prior to being invited to participate in the study. Participants came into the laboratory for a visit where they met with a trained research assistant and completed informed verbal and written consent. Once inclusion or exclusion criteria were confirmed, participants completed an in-clinic assessment. The research assistant then provided the participant with an Android smartphone and trained them on how to use the Ethica Data app [31]. At the end of the study participation, participants were compensated.

### Measures

#### EMA

Participants were asked to respond to prompts from the Ethica app 3 times per day for a period of 7 days. Surveys were administered a minimum of 90 minutes apart throughout each day at randomized time points within the windows of 10 AM-1 PM, 2-5 PM, and 5-8 PM. Participants were asked a variety of questions related to emotional experience and social context. Questions pertaining to emotional experience included a 6-point (“Not at all,” “Slightly,” “Somewhat,” “Moderately,” “Very much,” and “Extremely”) scale for happiness, sadness, and anxiety (see Table 1 for EMA questions and forced-choice responses).

**Table 1:** Description of EMA^a^ prompts and response choices.

EMA prompt	Response choices
How (happy or sad or anxious) are you feeling right now? How much are you interacting (ie, talking, playing) with the people around you? How much are you enjoying these interactions?^b^	Not at all Slightly Somewhat Moderately Very Much Extremely
How well do you know this person?^b^	I do not know the people here I know these people a little I am close to these people These people are family or very close friends or partners
Since the last prompt, how many times did you talk or communicate with someone?^b^	No interaction 1 interaction 2 or 3 interactions 4 or more interactions

^a^EMA: ecological momentary assessment.

^b^Prompt was only provided if participants responded that they were with others at the time of the prompt.

#### Greenspace

We quantified greenspace by calculating the NDVI, a proxy measure of the density of vegetation in a given geographical area. NDVI is mathematically defined as the quotient of the near-infrared light reflected by vegetation (NIR) minus the visible (VIS) radiation and the NIR radiation plus the VIS radiation emitted:

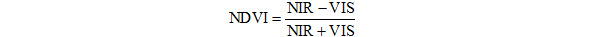



Satellites above the earth’s surface calculate NDVI values from the VIS and NIR light reflected by vegetation. Sparse vegetation reflects more VIS light and less infrared light, while dense vegetation absorbs most of the VIS light and thus reflects a large portion of the NIR light. The resulting calculated NDVI is a number that ranges from –1 to 1 where negative values represent water and values closer to 1 represent the highest levels of vegetation density. We excluded negative values from the analyses given limited data linking exposure to bodies of water and health outcomes. Geo-location data derived from smartphone sensors can be used to calculate NDVI values based on time spent in various locations, providing a fine-grained assessment of greenspace exposure within individuals as they go about their daily lives [32]. Geo-location data were collected at a sampling rate of 1 Hz every 6 minutes, which contained information about longitude, latitude, accuracy, and time stamps.

#### Ambient Audio

Study phones were also programmed to semirandomly record 5-minute intervals of ambient audio for every 30 minutes throughout the study period. This method has shown evidence of acceptability and feasibility in SSD [28]. Participants wore a visible button, approximately 3 inches in diameter, alerting bystanders that conversations may be recorded. To reduce social desirability bias, participants were not informed of times that they were actively being recorded.

We limited audio transcription to only those instances in which the user was engaged in an interpersonal exchange. We defined social interactions as reciprocal, tangible user speech containing content intended for the purpose of communication with another human. A new social interaction was coded at times when a participant engaged in conversation with a different person or after a lapse in speech for twenty seconds or longer. Given this definition, we excluded speech that (1) did not involve the participant, (2) was directed toward animals or pets, (3) was directed toward infants, (4) involved singing (either to or without music), or (5) was designated as self-talk. Unintelligible speech was also omitted.

Audio recordings were imported into Audacity (The Audacity Team) [33], a free, open-source computer software with audio playback and editing capabilities. Audacity includes features that enhance the user listening experience and help augment facets of the audio transcription process, such as visualizing audio tracks, amplifying and normalizing audio, and manipulating playback speed. Audio recordings were valid and subsequently included if a file opened and had audio data. In this study, trained graduate and undergraduate research assistants transcribed audio data. Discrepancies or questions regarding transcriptions were resolved during weekly consensus meetings. We chose to use human transcription over automated services to ensure social interactions were accurately identified, as existing automated speech recognition software is limited in its capabilities.

### Ethical Considerations

The study underwent a full review by the Boston University Charles River Campus Institutional Review Board (Protocol #: 4394). All participants provided informed consent before study participation, completed baseline questionnaires, and installed the Ethica platform on provisioned devices.

### Statistical Analysis

#### Greenspace Exposure

**Figure 1:**
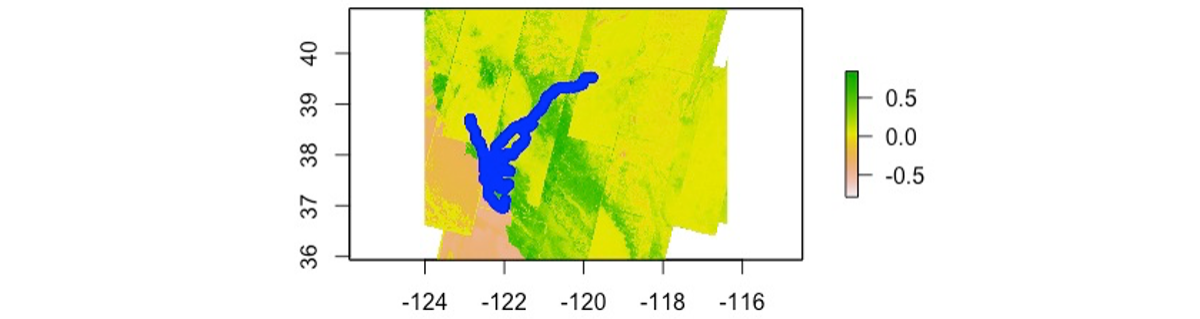
Map of geo-location and corresponding greenspace exposure (normalized difference vegetation index [NDVI]) of all participants (n=34). Lower numbers represent areas of less dense vegetation and higher number represent more densely vegetated areas. Negative numbers are indicative of water.

A raster file of NDVI data (ranging from –1 to 1) for the San Francisco Bay area was extracted from the USGS Landsat8 Collection 1 Tier 1 TOA Reflectance from Google Earth Engine. This image was optimized to include the date of participant involvement in the study as well as least amount of cloud coverage. GPS data were preprocessed to exclude network-passive and GPS-passive coordinates. Since we did not obtain information on the participants’ addresses we manually tagged “home” by taking the most frequent GPS coordinate pair between 9 PM and 6 AM [27]. These coordinates were then removed from the data set to exclude the NDVI values when they were at home. All GPS coordinates were linked to applicable NDVI values and then further processed to exclude negative NDVI values (indicating the participant was in or close to water; see Figure 1). We then calculated the daily mean NDVI for each participant and, due to the nonnormality of the data distribution (including significant positive skew), we log-transformed the values. One participant was excluded from the analysis due to cross-country travel during the study period. For participants who temporarily left the San Francisco metro area during the study period, we ran a sensitivity analysis that excluded data from the days on which they left the area. In multilevel models of daily associations between affect (EMA and speech) and greenspace exposure, results remained unchanged in this analysis. Thus, all data points were included in the analyses reported in this paper. In addition, we conducted a sensitivity analysis where we controlled for time spent at home. This analysis presented the same pattern of findings in multilevel models of daily associations between affect (EMA and speech) and greenspace exposure. Thus, results presented below are not adjusted for time spent at home.

#### Speech in Ambient Audio

Speech transcriptions were analyzed using Linguistic Inquiry and Word Count (LIWC) [34]. LIWC is a computerized software that processes over 6400 words or word stems across more than 90 categories. For this study, we focused on total word count, positive affect words (eg, “happy”), and overall negative affect words, which include the subcategories anxiety (eg, “worried”), anger (eg, “annoyed”), and sadness (eg, “crying”). While LIWC also categorized other negative words not captured by those subcategories (eg, “hurt,” “ugly”), we did not include these words in the analyses. LIWC calculates percentage scores for each category to account for the total words spoken in a sample. Speech transcriptions adhered to LIWC protocol, which contains specific procedures that ensure the lexical analysis software can reliably process data.

We examined all data for assumptions of planned statistical analyses (eg, homogeneity of variance and normality of data distributions). We aggregated repeated assessments of affect on the daily level to create a mean value for each variable. Rating of positive and negative affect, as well as ambient audio variables, were not normally distributed. As such, we examined group differences for demographic, NDVI, affect experience, and LIWC metrics using Wilcoxon Rank Sum tests. We conducted Spearman correlations to examine associations between NDVI, EMA-reported affect, and speech-based word counts and categories. We examined group differences in variables (eg, demographics and clinical assessments) and ambulatory data (ie, EMA, audio, and NDVI). In addition, we ran bivariate correlations to evaluate how relevant demographic variables (eg, age and years of education) were associated with NDVI. Given the exploratory nature of the analyses, we considered Spearman correlation magnitudes of 0.30 or greater (moderate effect) as meaningful signals. We followed up bivariate correlations that were at least moderate in magnitude with multilevel models conducted using Mplus (Muthen and Muthen). Two-level models were conducted to examine relationships between EMA-reported affect and NDVI within subjects at the daily level. All models involved individual-level clustering and person mean-centered NDVI values to examine deviations in the means at the individual level. We present standardized estimates of coefficients in the results. Two-tailed *P* values less than .05 were considered statistically significant.

## Results

### Overview

**Figure 2:**
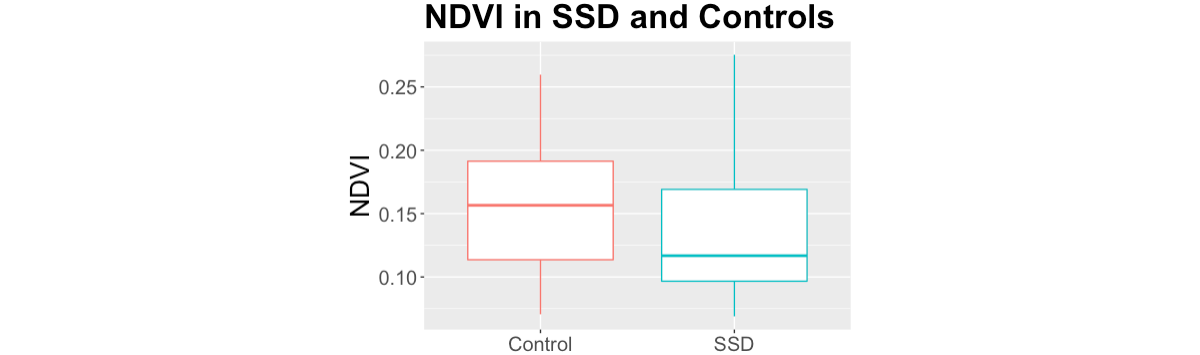
Greenspace exposure (NDVI) by group. Lines represent the median within each group throughout the duration of the study period. NDVI: normalized difference vegetation index; SSD: schizophrenia spectrum disorder.

People with SSD were older and less likely to be employed than controls (see Mote et al [35], for full demographic information). Groups did not significantly differ in mean EMA-reported positive (*P=*.06) or negative affect (*P*=.16), or LIWC total word count (*P*=.09). People with SSD used significantly more positive affect words (*P*=.04) and fewer anger words (*P*=.04) than controls. Groups did not significantly differ in overall NDVI exposure throughout the duration of the study (*P*=.59; see Figure 2) or mean hours spent at home (*P=*.84; Table 2). There were also no significant associations between demographic variables and greenspace exposure within groups (see Table 3).

**Table 2:** Study demographics.

		SSD^a^	Controls
Age (years), mean (SD)		53.30 (7.70)	44.07 (14.38)
Years of education, mean (SD)		14.06 (3.02)	15.36 (3.30)
**Gender, n (%)**			
	Male	15 (75)	11 (79)
	Female	5 (25)	3 (21)
**Employment status, n (%)**			
	Unemployed	11 (44)	3 (21)
	Employed	5 (25)	11 (79)
	Not reported	4 (16)	N/A^b^

^a^SSD: schizophrenia spectrum disorder or schizoaffective disorder.

^b^N/A: not applicable.

**Table 3:** Group differences in EMA^a^ and LIWC^b^ variables.

	SSD^c^, mean (SD)	Controls, mean (SD)	*P* values
EMA happy	2.66 (1.27)	3.22 (1.03)	.14
EMA sad	0.79 (0.91)	0.5 (0.79)	.06
EMA anxious	0.95 (0.93)	0.70 (0.73)	.25
LIWC word count	734.72 (1135.62)	1434.25 (1596.62)	.09
LIWC positive affect words	5.74 (3.88)	4.10 (3.20)	.04
LIWC negative affect words	0.99 (1.28)	1.34 (1.14)	.07
Anxious words	0.08 (0.16)	0.13 (0.35)	.50
Anger words	0.33 (0.55)	0.77 (1.09)	.04
Sad words	0.22 (0.46)	0.13 (0.14)	.40
NDVI^d^	0.14 (0.06)	0.14 (0.07)	.59
Time spent at home (h)	16.3 (5.21)	15.7 (6.83)	.84

^a^EMA: ecological momentary assessment.

^b^LIWC: Linguistic Inquiry and Word Count.

^c^SSD: schizophrenia or schizoaffective disorder.

^d^NDVI: normalized difference vegetation index.

### Greenspace Exposure (NDVI) and EMA-Reported Affect

In both groups, there were small to moderate associations between greater average greenspace exposure and lower average EMA-reported negative affect (sadness and anxiety) across the study (rho values between –0.22 and –0.32; see Table 4). When examining associations at the daily level using multilevel models, higher greenspace exposure on a given day was associated with significantly lower EMA-reported anxiety on that day among controls (*b*=–0.40, *P*=.03, 95% CI –0.76 to –0.04); there were no significant associations between daily greenspace exposure and EMA-reported happiness (*b*=0.68, *P*=.31, 95% CI –0.63 to 2.0) or sadness (*b*=0.04, *P*=.85, 95% CI –0.39 to 0.47) in controls. Among people with SSD, there were no significant associations between greenspace exposure and EMA-reported affect at the daily level, including anxiety (*b*=–0.37, *P*=.21, 95% CI –0.94 to 0.21), happiness (*b*=0.06, *P*=.89, 95% CI –0.77 to 0.88), or sadness (*b*=–0.31, *P*=.43, 95% CI –1.07 to 0.46).

**Table 4:** Associations (Spearman ρ) between demographics, affect, and greenspace exposure across the study.

		Correlation with greenspace exposure (NDVI^a^)	
		SSD^b^	Controls
**Demographics**			
	Age	–0.12	–0.09
	Years of education	<0.01	0.28
**EMA^c^-reported affect**			
	Happy	–0.22	0.12
	Sad	–0.05	–0.28
	Anxious	–0.25	–0.23
**Affect expressed in speech (LIWC^d^)**			
	Positive affect words	<–0.01	-0.19
	Negative affect words	–0.29	0.02
	Anxious words	–0.26	–0.13
	Anger words	–0.37	–0.04
	Sad words	–0.16	0.21

^a^NDVI: normalized difference vegetation index.

^b^SSD: schizophrenia or schizoaffective disorder.

^c^EMA: ecological momentary assessment

^d^LIWC: Linguistic Inquiry and Word Count.

### Greenspace Exposure (NDVI) and Affect-Expression in Speech (LIWC)

In controls, overall greenspace exposure was generally unrelated to affect expression in speech (see Table 4). At the daily level, greater NDVI was associated with a significantly higher proportion of negative affect words spoken (*b*=0.71, *P*=.01, 95% CI 0.17-1.25), but not with positive affect words spoken (*b*=–0.66, *P*=.27, 95% CI –1.85 to 0.52). At the daily level, greater NDVI was associated with a significantly higher proportion of anger words (*b*=0.66, *P*<.001, 95% CI 0.29-1.04), but not sadness words (*b*=0.04, *P*=.85, 95% CI –0.39 to 0.47), spoken. In people with SSD, there was a moderate association between greater overall greenspace exposure and a lower proportion of negative affect words spoken, including anxious and anger words specifically (see Table 4). The daily associations between greenspace exposure and positive (*b*=0.32, *P*=.90, 95% CI –4.65 to 5.30) or negative affect (*b*=–0.82, *P*=.07, 95% CI –1.69 to 0.06) words spoken in SSD participants were not statistically significant. At the daily level, greater NDVI was not associated with the proportion of anger words (*b*=–0.05, *P*=.77, 95% CI –0.34 to 0.25) or sadness words (*b*=–0.31, *P*=.43, 95% CI –1.07 to 0.46) spoken.

## Discussion

### Principal Results

In this study, we examined the association between greenspace exposure and affect among people with and without SSD. We incorporated active (EMA) and passive (audio samples of speech and GPS geo-location) smartphone measures of both greenspace and affect experience and expression. In both groups, greater greenspace exposure showed small to moderate associations with lower EMA-reported negative affect, while greenspace was unrelated to positive affect.

Participants with SSD who were exposed to greater levels of greenspace reported lower anxiety and sadness throughout the study. They also demonstrated a lower proportion of negative affect words spoken. Thus, for people with a serious mental illness, exposure to greenspace may be beneficial to well-being. Though, as noted, these associations were relatively small and were not present at the daily level. As such, there may not be a dynamic association between greenspace exposure and affect, at least over a relatively brief period of time. Instead, lower negative affect and greenspace exposure may covary at the between-person level (ie, those more likely to be around greenspace generally experience less negative affect).

Controls with greater exposure to greenspace throughout the study similarly experienced a lower negative affect on average. Furthermore, momentary analyses demonstrated that controls reported lower anxiety on days with higher greenspace exposure. Thus, evidence of covariation between changes in greenspace exposure and affect experience was more robust in controls than participants with SSD. It could be that controls were more likely to experience benefits of greenspace exposure than those with SSD, or that the groups differed in the types of greenspace, or the ways in which they interacted with these spaces, leading to differential outcomes. Future research can address these questions by characterizing the quality of greenspace exposure using supplemental methods (eg, additional EMA questions or more fine-grained NDVI assessment).

Interestingly, controls spoke a greater proportion of negative affect words on days in which they had greater greenspace exposure. Although this association between greenspace exposure and negative affect expression was contrary to our hypothesis, it is common to see discrepancies between affect experience and expression [36]. That is, what one experiences internally often does not correspond with what they demonstrate in verbal or nonverbal expression. In the context of this study, control participants may have experienced less anxiety on days with more greenspace exposure, but the higher frequency of negative affect words on these days did not correspond with their experience.

Relatedly, it is important to note the limitations in inferring emotion from speech using the LIWC; this method identifies the content of speech but does not consider the context in which those words are spoken. Without additional context, types of words used may not accurately represent the situation, or could even represent the opposite situation (eg, “I feel so much less anxiety here outside!”). The person speaking could also be reflecting on things other than their own affect (eg, “My boss seemed so angry this morning”). Future research should focus on independent and interacting relationships between experienced and expressed affect of greenspace exposure.

While some results were in line with our hypotheses, larger samples may be necessary to identify small to moderate associations between greenspace exposure and affect in participants with SSD at the daily level. Additionally, groups did not differ in overall greenspace exposure across the study, which may be due quality not quantity of the greenspace exposure. For example, a park in the middle of a noisy, busy city might have different restorative benefits than a quiet, state park in the suburbs.

### Comparison With Prior Work

Our work contributes to a growing literature that aims to characterize the influence of greenspace on well-being. Similar to previous work, we did find that greater greenspace exposure was associated with lower anxiety in the general population [37,38]. However, unlike in prior work looking at greenspace in people with psychosis [29], we did not find group differences in mean greenspace exposure, which could be explained by differences in geographic location between the 2 studies. The site of our study, the San Francisco Bay Area, has relatively temperate weather, providing more opportunities for regular outdoor activities, including in parks and other venues with adequate greenspace. Thus, access to greenspace may be less impacted by other key differences between the groups, such as transportation or other resources.

### Limitations

A primary limitation of this study is the small sample size, potentially limiting the power to detect modest relationships between greenspace exposure and affect in daily life. We also did not incorporate additional confounds (eg, activity level) that could influence the interpretation of the results, and will be a point of focus in future work. Although our design does provide rich, temporally dense data, it may be important to measure greenspace and affect over a longer period of time to detect dynamic associations. There are also limitations in the use of GPS-derived geo-location as a marker of NDVI (eg, the participants’ exact address, if they were outside or inside during a given timepoint, or what kind of greenspace they are being exposed to are all unknown). We also did not gather data related to subjective experience of greenspace exposure, which could serve to improve understanding of the degree to which participants valued or enjoyed such exposure, or their perception of degree of “greenness” of their environment. It is also important to note that not all greenspace is created equal; more objective and subjective information on the context surrounding greenspace exposure (eg, is the person in a small park in an urban environment, or in the middle of a lush conservation area?) could provide more robust modeling of associations with affect.

### Future Directions

More work is required to further understand the relationship between greenspace exposure and affect in those with and without SSD. Additional EMA questions could provide details on the participants’ surroundings (eg, are they physically outdoors or by a window) to fully understand their greenspace exposure. More questions related to affect experiences and mood could also help identify nuanced relationships between well-being and greenspace exposure. In future work, we can also test the momentary relationship between greenspace exposure and affect at a more temporally fine-grained level of analysis (eg, minutes or hours). Future directions can also harness other methods apart from LIWC to gather more rich information from speech. Some of these data science approaches include using machine learning algorithms to detect the affect of speech [39].

### Conclusions

Overall, our collection of both the active and passive smartphone data allowed for the assessment of within-person fluctuations in affect and their associations with greenspace exposure on a daily level in people with and without SSD. In both groups, greater greenspace exposure was associated with lower EMA-reported negative affect, but not positive affect, across the study. Within groups, controls reported lower anxiety on days with higher greenspace exposure, and also spoke a greater proportion of negative affect words, while people with SSD did not show associations at the daily level. Future studies should focus on additional aspects of greenspace exposure, affect, and experience on a fine-grained, dynamic level.

## Abbreviations

EMA: ecological momentary assessment
LIWC: Linguistic Inquiry and Word Count
NDVI: normalized difference vegetation index
NIR: near-infrared
SSD: schizophrenia or schizoaffective disorder
VIS: visible

## Data Availability

Data is available on Open Science Foundation at [40]. Given the sensitive nature of geo-location and audio files, we are unable to provide public access to these data.
